# Effects of a Short-Term High-Nitrate Diet on Exercise Performance

**DOI:** 10.3390/nu8090534

**Published:** 2016-08-31

**Authors:** Simone Porcelli, Lorenzo Pugliese, Enrico Rejc, Gaspare Pavei, Matteo Bonato, Michela Montorsi, Antonio La Torre, Letizia Rasica, Mauro Marzorati

**Affiliations:** 1 Institute of Molecular Bioimaging and Physiology, National Research Council, Segrate 20090, Italy; lorenz.pugliese@gmail.com (L.P.); michela.montorsi@unisanraffaele.gov.it (M.Mo.); letizia.rasica@ibfm.cnr.it (L.R.); mauro.marzorati@ibfm.cnr.it (M.Ma.); 2 Department of Neurological Surgery, Kentucky Spinal Cord Research Center, University of Louisville, Louisville, KY 40202, USA; enricorejc@kentuckyonehealth.org; 3 Department of Pathopysiology and Transplantation, Università degli Studi di Milano, Milano 20100, Italy; gaspare.pavei@unimi.it; 4 Department of Biomedical Sciences for Health, Università degli Studi di Milano, Milano 20100, Italy; matteo.bonato@unimi.it (M.B.); antonio.latorre@unimi.it (A.L.T.); 5 Department of Human Sciences and Promotion of Quality of Life, Telematic University S. Raffaele, Roma 00166, Italy; 6 Department of Psychology, Exercise and Sport Science Degree Course, Catholic University of the Sacred Heart, Milan 20100, Italy

**Keywords:** nitric oxide, oxygen cost of exercise, intermittent high-intensity exercise, diet

## Abstract

It has been reported that nitrate supplementation can improve exercise performance. Most of the studies have used either beetroot juice or sodium nitrate as a supplement; there is lack of data on the potential ergogenic benefits of an increased dietary nitrate intake from a diet based on fruits and vegetables. Our aim was to assess whether a high-nitrate diet increases nitric oxide bioavailability and to evaluate the effects of this nutritional intervention on exercise performance. Seven healthy male subjects participated in a randomized cross-over study. They were tested before and after 6 days of a high (HND) or control (CD) nitrate diet (~8.2 mmol∙day^−1^ or ~2.9 mmol∙day^−1^, respectively). Plasma nitrate and nitrite concentrations were significantly higher in HND (127 ± 64 µM and 350 ± 120 nM, respectively) compared to CD (23 ± 10 µM and 240 ± 100 nM, respectively). In HND (vs. CD) were observed: (a) a significant reduction of oxygen consumption during moderate-intensity constant work-rate cycling exercise (1.178 ± 0.141 vs. 1.269 ± 0.136 L·min^−1^); (b) a significantly higher total muscle work during fatiguing, intermittent sub-maximal isometric knee extension (357.3 ± 176.1 vs. 253.6 ± 149.0 Nm·s·kg^−1^); (c) an improved performance in Repeated Sprint Ability test. These findings suggest that a high-nitrate diet could be a feasible and effective strategy to improve exercise performance.

## 1. Introduction

Nitric oxide (NO) is a gaseous signaling molecule linked to a variety of physiological functions in mammalian cells, including the regulation of blood flow, mitochondrial biogenesis, excitation–contraction coupling, calcium handling, oxidative stress, and skeletal muscle repair [1]. NO is produced endogenously via the l-arginine-NO pathway by the nitric oxide synthase (NOS) enzymes in nervous tissue, the cardiovascular system (by the endothelium), and skeletal muscle [2,3]. However, an alternative source of NO has recently been described [4]. In fact, inorganic nitrate (NO_3_^−^), ingested from dietary sources (e.g., beetroot) or pharmacologic compounds (e.g., sodium/potassium nitrate), can be reduced in vivo to nitrite (NO_2_^−^) and subsequently converted to NO by numerous NO_2_^−^ reductases [5].

A growing body of evidence demonstrates that acute (2–3 h) and short term (3–6 days) pharmacological (e.g., sodium/potassium nitrate) or dietary (e.g., beetroot juice) NO_3_^−^ supplementation reduces whole body oxygen cost during moderate-intensity exercise, and improves exercise tolerance—at least in sedentary or moderately-trained subjects [6,7,8,9,10,11,12]. Given that the alternative NO_3_^−^–NO_2_^−^–NO pathway seems to increase NO bioavailability, especially in an acidic environment and in relatively hypoxic tissues [13], human studies have also been conducted in order to assess whether NO_3_^−^ supplementation positively affects muscle contraction properties at higher exercise intensities, when muscle PO_2_ and pH decline to a greater extent [14].

On the other side, it has been observed that maximal voluntary or involuntary (electrically evoked) isometric contraction, force–frequency relationship, and fatigability of quadriceps muscles are substantially unchanged following 4 days [15], 7 days [16], or 15 days [17] of NO_3_^−^ supplementation. At the same time, nitrate supplementation has unclear effects on high-intensity intermittent activities. Although some studies have found an improvement of high-intensity intermittent performance following NO_3_^−^ supplementation [18,19,20,21], others have failed to observe a positive effect [22,23]. The reasons for these controversial results may be attributed to differences in duration and dose of the supplementation scheme, in the exercise protocols employed [24], and in the individual aerobic fitness level of the participants.

So far, most of the studies that have investigated the ergogenic effects of dietary nitrate supplementation have used either beetroot juice or sodium nitrate. To our knowledge, only one study has evaluated the effects of a dietary intervention (acute whole baked beetroot assumption) on exercise performance [25]. Since recent studies have demonstrated that a high-nitrate vegetable diet can increase plasma NO_3_^−^ and NO_2_^−^ concentrations to similar level of beetroot or nitrate salt ingestion [26,27], this form of NO_3_^−^ supplementation could represent an alternative dietary intervention able to positively affect exercise performance.

The aim of this study was to test the hypothesis that a diet containing NO_3_^−^-rich vegetables increases plasma NO_3_^−^ and NO_2_^−^ concentrations and positively influences exercise performance. In particular, we expect that a diet high in NO_3_^−^ can reduce the oxygen cost of exercise at moderate intensity and increase muscle performance during high-intensity intermittent activities.

## 2. Materials and Methods

### 2.1. Subjects

In this randomized crossover study, seven healthy males recreationally involved in basketball, badminton, and futsal (mean ± SD; age, 25 ± 2 years; body mass, 66.3 ± 6.0 kg; height, 1.74 ± 0.05 m) volunteered to participate in this study. Considering the standard deviations (SDs) of circulating nitrate levels based upon our previous findings [11], this *n* value allowed the detection of significant differences between groups (if present) with an alpha level of 0.05 and a beta level of 0.20 (Prism 6.0, GraphPad Software, La Jolla, CA, USA). Before the start of the study, participants underwent a complete medical screening (medical history, physical examination, and resting electrocardiogram) to ensure that there were no contraindications to study participation. An incremental cycle ergometer test up to exhaustion was also performed for the determination of peak oxygen consumptio ($\dot{V}O_{2}$ peak) and peak work rate (41.2 ± 4.7 mL·kg^−1^·min^−1^; 226 ± 49 W). All subjects gave their written informed consent to participate after the experimental procedures, associated risks, and potential benefits of participation had been explained. All procedures were in accordance with the recommendations found in the Declaration of Helsinki (2000) of the World Medical Association.

### 2.2. Study Design

Individual subjects’ diet was recorded during a 7 day period, before the experimental phase. NO_3_^−^ usual intake was estimated considering vegetable and fruit intake, according to reference tables [28]. Then, a nutritionist elaborated two diet schemes: a control diet (CD), with a NO_3_^−^ intake similar to that usually ingested, and a diet with a high nitrate intake (HND). HND and CD diets were iso-energetic (about 2200 kcal) in accordance with subjects’ habitual energy intake and matched to physical activity levels, and they contained a similar distribution of macronutrients (55% carbohydrates, 15% proteins, 30% fats), except for nitrate [29,30]. Fruits and vegetables ensured the different NO_3_^−^ intake (Table 1).

The intake of NO_3_^−^ corresponded to ~8.2 mmol∙day^−1^ and ~2.9 mmol∙day^−1^ in HND and CD, respectively. Subjects were invited to follow the diet scheme for 6 days and were evaluated at days 5 and 6. A 20 day washout period separated the two interventions. Subjects were instructed to strictly respect the nutritionists’ indications. Participants were not informed about the aims of the study and were led to believe that both interventions may be beneficial on exercise performance. Subjects were also required to abstain from using antibacterial mouthwash and chewing gum, as these are known to alter the oral bacteria responsible for the reduction of NO_3_^−^ to NO_2_^−^ [31].

### 2.3. Experimental Overview

All tests were performed at the same time of the day (±1 h). Subjects were instructed to arrive at laboratory about 3 h postprandial and to avoid caffeine and alcohol intake and strenuous exercise in the 24 h preceding each testing session. Subjects visited the laboratory on two consecutive days at the end of both HND and CD.

On day one, subjects performed one repetition of a moderate-intensity constant work rate cycling exercise. Measures of maximal voluntary torque and total muscle work—estimated as the sum of impulses generated during fatiguing intermittent sub-maximal knee extensions—were also recorded (see below for further details). On day two, a second repetition of the moderate-intensity constant work rate cycling exercise was performed. After 30 min of rest, a cycling Repeated Sprint Ability test (RSA) was carried out (see below for further details). Prior to data collection, subjects were fully familiarized with exercise testing procedures.

### 2.4. Exercise Tests

**Moderate intensity constant-work rate cycling exercise**. An electromagnetically-braked cycle ergometer (Corival; Lode, Groningen, The Netherlands) was utilized. Subjects exercised at their freely chosen pedal frequency (80 ± 5 rpm). Each subject performed two repetitions of a 6-min constant work rate moderate-intensity exercise (CWR). Transitions from unloaded pedaling to the imposed work rate were attained in ~3 s. The work rate was chosen to correspond to 50% of peak work rate reached during the incremental test.

**Isometric knee extensions**. Subjects were seated on a special chair, secured by a safety belt tightened around the shoulders and abdomen, with the arms grasping handlebars and the legs hanging vertically down. A strap was tightened around the subject’s dominant ankle, and was linked by a steel chain to a fixed frame. The chain length was regulated to obtain a knee angle of 110 degrees. The fixed frame was positioned behind the ankle to perform the isometric knee extensions. Subjects began the experimental session by performing a warm-up, which consisted of 20 sub-maximal isometric contractions at a self-selected intensity. After that, they performed two exercises in the same experimental session with the dominant lower limb only:

(A) Maximal voluntary contraction (MVC): subjects were asked to perform three MVCs of three-to-four seconds in duration each. To prevent fatigue, after each contraction subjects rested for two minutes. The highest force was multiplied by the moment arm in order to calculate maximal voluntary torque (MVT).

(B) Fatiguing intermittent submaximal knee extension: based on pilot studies, subjects performed intermittent isometric knee extensions of 3.5 s, with 10 s of rest between them. The target torque to reach and maintain during each contraction was set at 75% of the actual MVT (i.e., at the same relative intensity, in HND and CD). Two different auditory feedbacks were given to subjects: (1) a “ring”, preceded by a countdown, determined the start and the end of each contraction; (2) a monotonic sound highlighted the reaching of the torque target level. Experimental sessions ended when subjects were not able to reach the target torque for two consecutive contractions.

**Repeated Sprint Ability test (RSA)**. RSA consisted of five “all out” 6-second sprints on a cycle ergometer (894E, Monark Exercise AB, Vansbro, Sweden) separated by 24 s of inactive recovery [32]. Subjects pedaled in a seated position, and the mechanical resistance (F) was set at 0.74 N∙kg^−1^ body mass.

### 2.5. Measurements

**Physiological variables**. Pulmonary ventilation ($\dot{V}E$), $\dot{V}O_{2}$, and carbon dioxide output ($\dot{V}CO_{2}$) were determined breath-by-breath by a computerized metabolic cart (Vmax29c; SensorMedics, Bilthoven, The Netherlands). Heart rate (HR) was determined from the electrocardiogram signal. Gain values (G)—the variable estimating the O_2_ cost of cycling—were calculated as Δ$\dot{V}O_{2}$ ($\dot{V}O_{2}$ at the end of CWR minus resting $\dot{V}O_{2}$) divided by work rate. Blood lactate concentration ([La]_b_) was measured at rest and at several times during recovery on 20 µL of capillary blood obtained from a pre-heated earlobe by an enzymatic method (Biosen C-line; EKF Diagnostics GmbH, Barleben, Germany). The highest [La]_b_ was taken as [La]_b_ peak.

**Force recording**. A force sensor (TSD121C, BIOPAC Systems, Inc., Goleta, CA, USA) was connected in series to the chain, which connected the fixed frame of the special chair to the strap tightened around the subject’s right ankle. Force analog output was sampled at a frequency of 1 kHz using a data acquisition system (MP100, BIOPAC Systems, Inc., Goleta, CA, USA) connected to a personal computer by means of an USB port.

**Surface Electromyography (EMG) recording**. EMG data were collected from the right (dominant) thigh: vastus lateralis (VL) was selected as the main knee extensor muscle. Pre-gelled surface EMG electrodes (circular contact area of 1 cm diameter, BIOPAC Systems, Inc., Goleta, CA, USA) were placed (inter-electrode distance equal to 20 mm) at two-thirds on the line from the anterior spina iliaca superior to the lateral side of the patella [33]. In order to ensure a good electrode–skin interface, prior to the application of the electrodes, the subject’s skin was shaved, rubbed with an abrasive paste, and cleaned with a paper towel. EMG electrodes were placed at the beginning of the experimental session, and were not removed between the two exercises. The locations of the electrodes during the first experimental session were marked on the skin with a permanent ink pen. In order to place electrodes in the same positions prior to the second session, the subjects were asked to refresh these contours daily. To record the EMG data, the electromyography system (EMG100C, BIOPAC Systems, Inc., Goleta, CA, USA; Low Pass Filter: 500 Hz; High Pass Filter: 10 Hz; Noise Voltage (10–500 Hz): 0.2 µV (rms); Zin: 2 M ohm; CMRR: 110 dB) was used. EMG data were sampled at a frequency of 1 kHz using a data acquisition system (MP100, BIOPAC Systems, Inc., Goleta, CA, USA), and processed using the program LabChart 7 Reader (ADInstruments Pty Ltd., Bella Vista, NSW, Australia).

**Force and EMG analysis**. As for MVC, a 500 ms window was centered at the maximal force exertion to calculate MVT (see above) and to analyze the surface electromyography (sEMG), its intensity being quantified by root mean square (RMS). During intermittent submaximal isometric contractions, no mechanical work is performed, so the torque-time integral (TTI) was used to estimate muscle work [34]. For each single knee extension, TTI and RMS of vastus lateralis (RMS-VL)—expressed as a percentage of maximal voluntary contraction (%MVC)—were calculated. In addition, the average value of RMS-VL calculated over the first three knee extensions was compared to the one obtained during the last three knee extensions, in order to investigate the fatigue effect on muscle activation (adapted from Mulder et al., 2007) [35].

**Power recording**. The power (P) values were calculated as P = F × d × RPM, where F is the resistance set (0.74 N∙kg^−1^ body mass), d is the distance covered by the flywheel at each revolution, and RPM is the number of revolutions per minute. Instantaneous P values were sampled at 50 Hz and then averaged each second. Peak Power (PP) was considered the maximal value of power recorded over a second.

**Blood sampling**. Resting blood samples were collected to determine plasma levels of nitrate and nitrite before the experimental phase and on day 6 of both diet periods, at least 2.5 h after the last meal. Venous blood was drawn from the antecubital vein into a 5-mL EDTA Vacutainer tube (Vacutainer, Becton, Dickinson and Company, Franklin Lakes, NJ, USA). Plasma was immediately separated by centrifuge (5702R, Eppendorf, Hamburg, Germany) at 1000× *g* for 10 min at 4 °C. Plasma samples were then ultrafiltered through a 10 kDa molecular weight cut-off filter (AmiconUltra; Millipore, EMD Millipore Corporation, Billerica, MA, USA) using a ultracentrifuge (4237R, ALC, Milan, Italy) at 14,000× *g* for 60 min at 4 °C to reduce background absorbance due to the presence of hemoglobin. The ultrafiltered material was recovered and used to measure nitrite and nitrate concentration by the Griess method using a commercial kit (Cayman, BertinPharma, Montigny le Bretonneux, France). Samples were read by the addition of Griess reagents at 545 nm by a microplate reader spectrophotometer (Infinite M200, Tecan Group Ltd., Männedorf, Switzerland). A linear calibration curve was computed from pure nitrite and nitrate standard. All samples were determined in duplicate, and the inter-assay coefficient of variation was in the range indicated by the manufacturer.

### 2.6. Statistics

Data were expressed as mean ± SD. A paired *t*-test was performed on all tests data to compare HND and CD. A two-way ANOVA for repeated measures with Bonferroni correction was applied when multiple comparisons were made. The significance level was set at *p* < 0.05. Statistical analysis was performed by a software package (Prism 6.0; GraphPad Software, La Jolla, CA, USA).

## 3. Results

### 3.1. Nitrate and Nitrite Plasma Levels

Before the experimental phase, plasma NO_3_^−^ and NO_2_^−^ concentrations were 24 ± 8 µM and 118 ± 32 nM, respectively. Following CD, plasma NO_3_^−^ and NO_2_^−^ concentrations were 23 ± 10 µM and 240 ± 100 nM, respectively, and not statistically different. After HND, plasma NO_3_^−^ and NO_2_^−^ concentrations significantly increased to 127 ± 64 µM and 350 ± 120 nM, respectively.

### 3.2. Moderate-Intensity Constant Work Rate Cycling Exercise

Mean values of the main physiological variables determined during the last ~30 s of CWR (carried out at the same absolute work rate in the two conditions) are presented in Table 2.

$\dot{V}O_{2}$ and $\dot{V}E$ values were significantly lower in HND vs. CD. The values of G (estimating the O_2_ cost of cycling) were significantly reduced by the high-nitrate diet (11.0 ± 1.2 vs. 13.3 ± 2.2 mL·min^−1^·W^−1^), even if they remained substantially closer to those usually observed in normal subjects (10 mL·min^−1^·W^−1^). Heart rate and blood lactate values were not different in the two conditions.

### 3.3. Isometric Knee Extension

**Figure 1:**
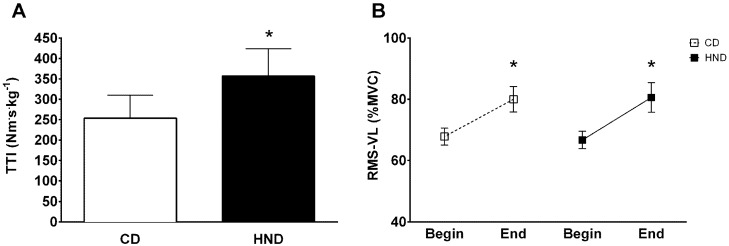
Knee extension fatiguing intermittent submaximal test. (**A**) Mean values (±SD) of total torque-time integral (TTI) and (**B**) root mean square of vastus lateralis (RMS-VL) recorded during the fatiguing intermittent submaximal test after control and high-nitrate diet. * *p* < 0.05.

MVT was not significantly different between HND (2.8 ± 0.5 Nm·kg^−1^) and CD (2.9 ± 0.6 Nm·kg^−1^). As for fatiguing intermittent submaximal exercise, after HND, the number of contractions performed was higher than after CD (47.1 ± 18.3 and 32.5 ± 12.4, respectively). The sum of TTI recorded was higher (*p* < 0.05) in HND (357.3 ± 176.1 Nm·s·kg^−1^) than in CD (253.6 ± 149.0 Nm·s·kg^−1^) (Figure 1A).

During the first three knee extensions (Begin), the average values of RMS-VL in both HND and CD (66.7 ± 7.6 %MVC and 67.8 ± 7.3 %MVC, respectively) were significantly lower compared to the average values of the last three contractions (End) (80.6 ± 12.7 %MVC and 80.0 ± 11.0 %MVC, respectively) (Figure 1B). No differences in RMS-VL values were detected between HND and CD.

### 3.4. Repeated Sprint Ability (RSA)

**Figure 2:**
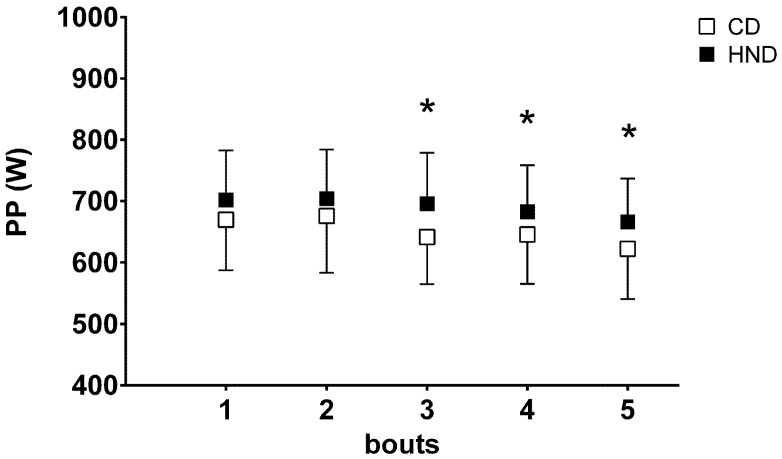
Repeated sprint ability test. Mean values (±SD) of peak power output (PP) obtained during the five bouts of the repeated sprint ability test (RSA) performed on a cycle ergometer after CD and HND. * *p* < 0.05.

There was no difference in absolute PP output (Figure 2) of the first two sprints between HND (701.9 ± 80.8 W and 704.2 ± 80.0 W) and CD (669.6 ± 81.8 W, 675.9 ± 92.1 W).

In contrast, the PP output of the 3rd, 4th, and 5th sprints were significantly higher in HND (696.0 ± 83.1 W, 682.5 ± 76.2 W, 666.1 ± 70.7 W, respectively) than in CD (641.4 ± 76.2 W, 645.6 ± 80.2 W, 622.2 ± 81.4 W, respectively). No significant difference in [La]_b_ during recovery was observed between the two conditions.

## 4. Discussion

In the present study, we examined the effects of a diet ensuring a high nitrate intake (by vegetables and fruits) on nitrate/nitrite plasma levels and exercise performance. Our results show that 6 days of a HND (~8.2 mmol∙day^−1^), compared to a CD (~2.9 mmol∙day^−1^), induced a significant rise of plasma nitrate and nitrite concentrations. These findings were associated with a reduced oxygen cost of aerobic exercise and an increased performance during high-intensity intermittent activities.

### 4.1. Plasma Nitrate/Nitrite Levels

Plasma nitrate concentration increased by ~500% and plasma nitrite concentration increased by ~50% after 6 days of HND, whereas no significant changes were observed after CD. The effects of HND in the present study are comparable to those reported in previous studies where supplementation with green leafy vegetables was pursued [26,36]. Moreover, the plasma nitrate and nitrite levels achieved are very close to those observed in studies that have used either beetroot juice [7] or sodium nitrate [11] as a supplement. Thus, our data indicate that a short-term diet containing nitrate rich vegetables significantly affects nitric oxide bioavailability. However, it should be noted that subjects were instructed to consume their meal about 3 h prior to exercise testing. Thus, this experimental design does not guarantee the exclusion of the combined chronic and acute effect of nitrate ingestion on plasma nitrate and nitrite concentrations [37].

### 4.2. Constant Work Rate Cycling Exercise

The increase of nitrate/nitrite plasma levels observed after high-nitrate diet was associated with a 7.2% reduction of oxygen consumption during a moderate intensity constant work rate exercise. This reduction was associated with lower pulmonary ventilation, whereas heart rate and blood lactate concentration were not influenced. In humans, Larsen et al. [4] first reported a lower oxygen cost during submaximal exercise following nitrate supplementation. Subsequently, this result has been confirmed by several randomized controlled trials in both healthy subjects (for a review, see [38]) and patients with chronic disease conditions that severely impair oxygen delivery and/or utilization, such as chronic obstructive pulmonary disease, heart failure, or peripheral arterial disease [39]. From these studies, it has been hypothesized that the underlying mechanisms of a reduced oxygen cost of exercise involve: (1) an enhanced mitochondrial oxidative phosphorylation efficiency, measured as the amount of oxygen consumed per ATP produced (P/O ratio) [3,6]; or (2) a reduced ATP cost of muscle force production due to a reduction in the ATP cost of cross-bridge cycling (actomyosin ATPase) and/or Ca^2+^ handling (Ca^2+^-ATPase) [8]. Although we did not investigate the molecular effect of an increased NO bioavailability in this study, the results indicate that a diet containing nitrate-rich vegetables can influence the oxygen cost of moderate intensity exercise and could represent a useful ergogenic intervention to improve exercise tolerance similarly to either pharmacological (e.g., NaNO_3_^−^ and KNO_3_^−^) or dietary (e.g., beetroot juice) NO_3_^−^ supplementations.

### 4.3. Intermittent High-Intensity Activities

In this study we observed similar values of maximal voluntary isometric force of the knee extensors after both high-nitrate and control diet. This result is consistent with the literature on the effects of nitrate supplementation (in the form of beetroot juice) on maximal voluntary force of young healthy physically active males [15,16,17]. At the same time, following HND, there was a significant improvement of muscle performance during both fatiguing submaximal knee extension exercise and Repeated Sprint Ability test. Indeed, HND resulted in an increased muscle work (higher number of muscle contractions and torque–time integral) during isometric knee extension and in an improved peak power output during the last bouts of the repeated sprint ability test. Although the present study did not evaluate specific muscular adaptations, the possible mechanisms underlying the induced enhancement in performance may involve several factors. A previous work in mice has shown an increase in myoplasmic free Ca^2+^ concentration, Ca^2+^ binding protein calsequestrin 1 and dihydropyridine receptors following several days of nitrate supplementation [40]. In humans, a reduced accumulation of intracellular phosphate ([P_i_]) during contraction has been documented following nitrate supplementation [8]. Finally, Fulford et al. [17] reported that 15 days of dietary nitrate supplementation reduces the phosphocreatine cost of force production during repeated isometric maximal voluntary contractions of quadriceps muscle. Thus, it should be hypothesized that after HND, subjects were able to perform greater muscle work according to an increased Ca^2+^ sensitivity and sarcoplasmatic Ca^2+^ release. Additionally, a higher restoration of phosphocreatine (PCr) during the recovery phases between the contractions may have occurred. It is known that after beetroot juice supplementation, PCr degradation is reduced and lower inorganic phosphate and adenosine di-phosphate have been observed, reflecting an enhanced fatigue resistance from metabolite accumulation. In this study, the occurrence of an improved contraction efficiency (a reduced PCr depletion to force production) and/or an enhanced excitation–contraction coupling [16,17] without different levels of fatigue is supported by EMG data. Indeed, RMS value recorded from vastus lateralis was significantly higher at the end of the knee extensions fatiguing protocol compared to resting condition, but it was similar following HND and CD. Finally, it must be acknowledged that the increased bioavailability of nitric oxide has been also related to an enhanced mitochondrial oxidative phosphorylation efficiency and—especially in fast twitch fibers—to an improved muscle perfusion [38,40]. Thus, we cannot exclude that an increased production of ATP for the same oxygen consumption, as well as an increase in muscular blood flow and oxygenation after HND, could have contributed to a reduction in metabolic perturbation [20,41].

Another interesting finding of the present study is that the Repeated Sprint Ability test was significantly improved after HND. Although there was no difference in Peak Power output developed during the first two sprints, following HND, the Peak Power output of 3rd, 4th, and 5th sprint was significantly higher than that recorded after CD. These results are in accordance with Thompson et al. and Aucouturier et al., who found an improved repeated sprint performance after NO_3_^−^ supplementation in team-sports players [20,21]. At the same time, these results are in contrast with other studies in which no effects on intermittent exercise performance after NO_3_^−^ ingestion were observed [22,23]. The differences may be related to several factors. Christensen et al., for example, observed that nitrate supplementation did not change either peak or mean power for all six 20 s sprints [22]. However, the subjects recruited for this study were highly trained cyclists (~70 mL·kg^−1^·min^−1^), and it has been demonstrated that subjects with a high level of aerobic fitness may not benefit from NO_3_^−^ supplementation [11,42]. Martin et al. did not find any beneficial effects on repeated sprint exercise after NO_3_^−^ ingestion [23]. Nevertheless, they had found an elevated standard deviation in subjects’ $\dot{V}O_{2}$max (49.6 ± 11.8 mL∙kg^−1^∙min^−1^), there was no information about plasma NO_3_^−^ and NO_2_^−^ concentrations after supplementation, and an active recovery was used between sprints. Finally, Buck et al. showed no effects on female athletes after acute NO_3_^−^ ingestion [43]; it is likely that an acute nitrate supplementation can be less consistent than a short term one [38]. Moreover, the potential ergogenic effects of nitric oxide bioavailability in females have to be fully understood [44].

### 4.4. Study Limitations

In the present study, the dose of 8.2 mmol∙day^−1^ of NO_3_^−^ in HND was chosen according to the most effective pharmacological or dietary (beetroot juice) supplementation regimes adopted in previous studies [3,7,8,9,11]. Although this amount is significantly higher than the estimated average nitrate intake of 1–2 mmol∙day^−1^ in the US and European populations, previous studies have utilized a similar nitrate intake as an effective intervention to reduce arterial hypertension [30,45]. This study shows that increasing daily dietary nitrate intake may also have important implications on exercise performance. We did not investigate possible adverse effects of our diet intervention, even if the subjects did not report any problem and the period of observation was quite short (6 days). However, these result need to be confirmed by further larger studies before reconsidering dietary recommendations. Furthermore, even if the two interventions differed from nitrate intake, we cannot exclude the possibility that other dietary compounds could be responsible for the performance changes.

## 5. Conclusions

In conclusion, this study has shown that the ingestion of nitrate-rich foods can increase plasma nitrate/nitrite concentrations and improve exercise performance. In particular, this nutritional intervention reduced energy demand during moderate-intensity exercise, enhanced muscle work during fatiguing intermittent submaximal contractions, and improved repeated sprint performance, whereas maximal isometric force or peak power output were not affected. These results suggest that a high-nitrate diet could be a feasible strategy for increasing plasma nitrate/nitrite levels and improving moderate intensity aerobic or high-intensity intermittent performance.

## Figures and Tables

**Table 1:** Dietary intake prescribed by the nutritionist for high-nitrate and control diet. The relative amounts of NO_3_^−^ content for servings are also shown. CD: control diet; HND: high-nitrate diet.

**CD**		
**Food**	**Approximate Amount for Daily Servings**	**NO_3_^−^ Content**
salad mix	180 g	2.4 mmol
broccoli	60 g	0.4 mmol
orange	150 g	0.0 mmol
cranberry juice	0.5 L	0.1 mmol
**HND**		
**Food**	**Approximate Amount for Daily Servings**	**NO_3_^−^** **Content**
raw spinach	40 g	4.8 mmol
cooked collard greens	80 g	3.2 mmol
banana	130 g	0.1 mmol
pomegranate juice	0.5 L	0.1 mmol

**Table 2:** Mean (±SD) values of the main respiratory, cardiovascular, and metabolic variables determined at the end of the moderate-intensity constant work rate exercise after high (HND) and control nitrate diet (CD). $\dot{V}O_{2}$: oxygen uptake; $\dot{V}CO_{2}$: carbon dioxide output; R: gas exchange ratio; $\dot{V}E$: pulmonary ventilation; [La]_b_: blood lactate concentration; HR: heart rate; * *p* < 0.05, significantly different between high and control diet.

	Work	$\dot{V}O_{2}$	$\dot{V}O_{2}$	$\dot{V}CO_{2}$	R	$\dot{V}E$	[La]_b_	HR
	W	L·min^−1^	mL·kg^−1^·min^−1^	L·min^−1^		L·min^−1^	mM	b·min^−1^
**CD**	74 ± 5	1.269 ± 0.136	18.9 ± 1.6	1.127 ± 0.118	0.89 ± 0.05	34.5 ± 3.6	5.15 ± 2.18	116 ± 17
**HND**	74 ± 5	1.178 ± 0.141 *	17.9 ± 2.8 *	1.049 ± 0.137 *	0.90 ± 0.05	33.0 ± 4.3	4.68 ± 1.84	112 ± 15
